# Recreational Cannabis Legislation: substance use and impaired driving among Canadian rural and urban postsecondary students

**DOI:** 10.1186/s42238-023-00175-y

**Published:** 2023-03-15

**Authors:** N’deye Rokhaya Gueye, Kevin Prada, Danielle de Moissac

**Affiliations:** Université de Saint-Boniface, Office #3217, 200 de la Cathédrale Ave., Winnipeg, MB R2H 0H7 Canada

**Keywords:** Canada, Recreational Cannabis Legislation, Substance use, Road safety, Emerging adult, Postsecondary student, Urban, Rural

## Abstract

**Background:**

Investigation of cannabis use trends among emerging adults (EA, aged between 18 and 24 years) following 2018 Canadian Recreational Cannabis Legislation (RCL) is critical. EAs report the heaviest cannabis use in Canada and are particularly vulnerable to the onset of problematic substance use.

**Objectives:**

To describe and compare post-RCL use of cannabis and other state-altering substances, as well as the prevalence of impaired driving, among EA postsecondary students in both rural and urban settings, studying on one of five campuses in either Manitoba, Ontario, or Quebec.

**Methods:**

For this quantitative cross-sectional study, a self-report survey was administered to 1496 EA postsecondary students in the months following RCL (2018–2019). Multiple logistic regression analyses were conducted to explore the influence of provincial and urban/rural living contexts on recreational cannabis use, other state-altering substance use and impaired driving behaviours, adjusting for sociodemographic variables.

**Results:**

Statistically significant differences were observed between cohorts in almost all measures. Quebec students were more likely to have consumed cannabis during their lifetime (AOR = 1.41, 95% CI [1.05, 1.90]) than all other cohorts. Rural cohorts all had greater odds of reporting consumption of cannabis during the previous year compared to urban cohorts (AOR = 1.32, 95% CI [1.04, 1.67]). However, the relation between cannabis use in the last month and operating a motor vehicle after using cannabis (lifetime and past month) and living context differed between subjects in Quebec and those in the two other provinces. Quebec’s students having lived mostly in urban contexts had greater odds of using cannabis in the past month and operating a motor vehicle after using cannabis (lifetime and past month) than those in rural contexts; the opposite was observed in Manitoba and Ontario. Differing interprovincial prohibitive/permissive legislation and licit cannabis infrastructure appeared to have little impact on post-RCL substance use.

**Conclusions:**

In Manitoba and in Ontario, rural/urban living context seems to better predict substance use and related road-safety practices, suggesting these trends supersede permissive/prohibitive provincial legislation and licit cannabis-related infrastructures. Further investigation into sociodemographic factors influencing state-altering substance use and impaired driving, and maintaining tailored cannabis misuse prevention campaigns, is warranted on Canadian campuses.

## Introduction

Federal Recreational Cannabis Legislation (RCL), enacted in Canada in October 2018, allows for the purchase and consumption of cannabis for recreational purposes (Government of Canada 2019). RCL has transformed the country’s substance use landscape, which continues to evolve in response to legislative, supply, and broader societal changes (Canadian Centre on Substance Use and Addiction 2019; Rotermann 2020; Smart and Pacula 2019). Interprovincial legislative disparities relating to licit cannabis distribution and use are observed (Legislative Assembly of Ontario 2018; Province of Manitoba 2018; Province of Quebec 2020). Differences according to rural and urban living context have also been reported concerning use of cannabis and of other state altering substances (Canadian Institute for Health Information 2019; Pirie and Simmons 2014). The impact of RCL on these disparities should be explored, particularly among at-risk populations such as young adults.

Today, emerging adults (EAs, 18 to 24 years of age) are the heaviest cannabis users in Canada (Statistics Canada 2019a, 2019b, 2019c) and, alarmingly, are most vulnerable to substance misuse (Arnett 2005). Reports on the impacts of prohibitive/permissive cannabis legislation on EA cannabis use and related behaviours are divided (Canadian Centre on Substance Use and Addiction 2019): while some conclude that legislation is inconsequential (Gueye et al. 2021; Midgette and Reuter 2020; Simons-Morton et al. 2010), others report modest post-RCL increases in consumption (Miller et al. 2017; Rotermann 2020). However, these realities have evolved and continue to do so, since RCL. Given also the paucity of research into geographical differences (considering both provincial and rural or urban contexts), especially among such at-risk groups as EAs and postsecondary students in Canada, cannabis use and related problematic road safety behaviours should be closely monitored, both at national and local levels. Only such targeted investigation can yield sufficient insights to develop effective prevention and intervention initiatives specific to geographical contexts. This paper discusses post-RCL cannabis use and impaired driving trends, as reported by EAs enrolled in five Canadian universities, allowing for interprovincial and rural/urban comparisons. Complementary substance use data, including alcohol and illicit drugs, are also discussed, as these provide additional insights into the potential impacts of RCL on EA substance use.

### Emerging adults at heightened risk for substance misuse and impaired driving

As they transition into adulthood, EAs continue to undergo complex processes of hormonal and neurological changes linked to healthy cognitive development and brain function (Laviolette 2019). The potentially deleterious and long-term effects of Δ-9-tetrahydrocannabinol (THC, cannabis’ main psychoactive compound) on the developing brain (Bossong and Niesink 2010; Rubino and Parolaro 2016) include the onset of psychological troubles (Hellemans et al. 2019; Laviolette 2019; Walters et al. 2018) and deficits in memory, attention, executive function (Wallingford et al. 2019), and emotional regulation (Laviolette 2019). EAs also tend to overestimate the prevalence of substance use among their peers, which may influence their own substance use (Arbour-Nicitopoulos et al. 2010; Derefinko et al. 2018). Furthermore, more permissive social norms and attitudes surrounding substance use have been found to predict greater substance use among EAs (Arbour-Nicitopoulos et al. 2010; Derefinko et al. 2018). Huỳnh et al. (2022), after finding negligible regional differences in substance use by 17- to 35-year-old populations throughout Canada, suggest that predictors for substance use may pertain more to individual differences, such as sensation seeking, impulsivity, and risk taking behaviours, rather than differences according to the substance used.

Given experimentation and establishment of lasting behaviours and attitudes being a staple of during this transitional period in their life (Patton et al. 2016; Wadsworth and Hammond 2019; Walters et al. 2018), EAs are particularly vulnerable to the onset and escalation of substance misuse (Arnett 2005) and impaired driving (Gueye et al. 2019; Perreault 2015). These observations are especially troubling considering the significant psychomotor inhibition triggered by cannabis use, which poses a salient threat to road safety. Post-cannabis collision risk has been shown to be greater among EAs compared to other Canadian age groups (Brubacher et al. 2020) and exponentially increases when alcohol and cannabis are used in tandem (Brubacher et al. 2020). This concern is substantiated by the knowledge that cannabis accounts for the most hospitalizations of Canadian youths aged 10 to 24 for harm caused by substance use (Canadian Institute for Health Information 2019). In fact, EAs, especially males, rate cannabis use as being less risky, and safer to drive while under its influence, than alcohol (Hellemans et al. 2019; Goodman et al. 2020). Males also exhibit lower assessment of risks associated with driving under the influence of cannabis compared to women (McDonald et al. 2021).

### Current trends in EA substance use and impaired driving

The evolving RCL context must also be taken into account when considering cannabis and substance-related problematic road safety behaviours among EAs. Although Brubacher et al.’s (2020) post-RCL review of impaired driving among younger drivers in Canada found the literature to be divided on the impacts of RCL on cannabis-impaired driving for younger demographics, this review did suggest that driving under the influence of cannabis may be more prevalent post-RCL for younger Canadians than driving after consuming alcohol. Inversely, however, 2019 national data representing the general Canadian population (independently from age) revealed that although alcohol-impaired driving was found to remain significantly more prevalent than drug-impaired driving following RCL, prevalence of both increased compared to the previous year, with drug-impaired driving increases significantly greater than alcohol-impaired driving (43% increase for drug-impaired, and 15% for alcohol-impaired driving nationally; Statistics Canada 2021). Given the potential harm caused by those driving while under the influence of state-altering substances, continued investigation into EA behaviours is vital.

EAs are not, however, a homogeneous population. Sociodemographic factors unrelated to legislation also seem to influence substance-related behaviour, including living in a rural or urban setting, as well as gender and age. Conflicting evidence on the association between rural/urban contexts and cannabis use is found in the literature: a 2014 Canadian study demonstrated greater prevalence of cannabis use among urban EAs (Pirie and Simmons 2014), whereas two American studies found no significant urban/rural difference in use for this population (Coughlin et al. 2019; Derefinko et al. 2018), and one Francophone High-School age study in Manitoba found rural living to be a significant predictor of alcohol use (Delaquis and de Moissac 2009). Notwithstanding, a 2018 study among Canadian 10- to 24-year-olds reported that hospitalization rates for harm caused by substance use was as much as 1.7 times higher for youths residing in rural or remote areas than peers residing in urban contexts (Canadian Institute for Health Information 2019). Taken together, these insights are of concern, as rural youths not only exhibit higher prevalence of substance use, but also face poorer access to substance-related prevention and treatment programmes than their urban peers (Canadian Institute for Health Information 2019). Canadian literature also reveals significant differences in substance use and outcomes depending on gender and age (Canadian Centre on Substance Use and Addiction, 2020; Gueye et al., 2019). For example, prevalence of hospitalization for harms caused by substance use among young women tends to peak in their mid-20s, whereas this trend continues to climb well into the 30s for young men (Canadian Institute for Health Information 2019). Men are also more likely to consume alcohol or cannabis (Hellemans et al. 2019; Pirie and Simmons 2014; Rotermann 2020; Statistics Canada 2019c) and to drive impaired, than females (Azagba et al. 2020; Rotermann 2020; Statistics Canada 2019a; Wallingford et al. 2019). However, a sense of belonging and connectedness to a larger community, positive relationships with adults, a sense of personal security, and rigorous family and peer supports are efficacious protective factors against substance misuse among this population (Tam 2018; Zuckermann et al. 2020). Considering these multiple factors of influence, compounded onto interprovincial RCL differences, it is not surprising to observe significant variations in cannabis-related behaviours (such as driving under a influence of cannabis) across Canada (Rotermann 2020). Given that EAs represent the bulk of undergraduate students in Canada, postsecondary populations are suitable candidates to gain insight into EAs’ prevalence of substance misuse and impaired driving. Although postsecondary students may exhibit fewer problematic substance-related behaviours than non-student EAs (Huỳnh et al. 2022), they remain an accessible and appropriate population within which to investigate this phenomenon.

### Provincial RCL contexts

Legislation regulating availability, sales, and public consumption of recreational cannabis differs between provinces. Initially, cannabis sales in Ontario and Quebec were only fulfilled online through the province’s Crown cannabis corporation via home delivery; retail locations were later introduced for in-person sales (Legislative Assembly of Ontario, 2018; Province of Quebec 2020). Manitoba adopted a different approach from the start: beyond online orders, multiple retail locations also opened upon RCL in Winnipeg, Manitoba’s capital and largest city, whereas the first retailer in Brandon, Manitoba’s second-largest city, opened one month later (Province of Manitoba 2018). Also, minimum legal age for cannabis consumption in Quebec was 18 years, while it was 19 years in Ontario and Manitoba at the time of this study (Quebec later raised legal age for consumption to 21 (Legislative Assembly of Ontario, 2018; Province of Manitoba 2018; Province of Quebec 2020)). Likewise, minimum legal age for alcohol consumption varied between provinces: the minimum legal age for alcohol consumption in Quebec and Manitoba is 18 years, while it is 19 years in Ontario. Callaghan’s search team has demonstrated that Canadian drinking age legislation has a significant impact on driving-related harms (Callaghan et al.^ 2014^; Callaghan, Gatley, Sanches, Asbridge 2014; Callaghan et al. 2016).

### Objectives

This paper reports a data subset from a broader 2018–2019 study (de Moissac et al. 2019) that investigated the mental health status and risk-taking behaviours of postsecondary students enrolled in five Canadian universities, in the provinces of Manitoba, Ontario and Quebec, representing various rural/urban and official language contexts. This paper’s objective is to describe and compare post-RCL use of cannabis, alcohol, and illicit drugs, and impaired driving, among postsecondary student EAs (18–24 years of age) in three provinces, comparing students who spent the majority of their life in a rural or urban. In light of the literature, authors hypothesized that students who spent the majority of their life in rural settings would be more susceptible to cannabis, alcohol, and illicit drug use, as well as to impaired driving, while also expecting to observe provincial differences in trends. Findings will serve policymakers, academic administrators, and healthcare providers seeking to design and implement targeted interventions promoting the health and wellbeing of postsecondary students across Canada and preventing hazardous driving practices, as the impacts of RCL continue to evolve.

## Methodology

### Design and procedure

A quantitative cross-sectional study was used to measure the sociodemographic profiles, substance use trends, and impaired driving behaviours, of postsecondary students between 18 and 24 years of age enrolled in undergraduate programmes in two universities in Manitoba (Université de Saint-Boniface (USB); Brandon University (BU)), one in Ontario (University of Ottawa (UO)), and two in Quebec (Université du Québec en Abitibi-Témiscamingue (UQAT); Bishop’s University (B’sU)). Most universities were selected for their similar size and contrasting rural/urban contexts, while also representing various official language realities (with teaching language being either English or French, Canada’s official languages) within majority/minority official linguistic contexts. UO was selected as it is Canada’s only bilingual (French and English) university. Following data collection, participants were assigned to one of six cohorts, one rural and one urban for each of the three included provinces, depending on which of these two contexts they reported having resided in for most of their life. Students were invited to participate in an online or paper-based survey, distributed on campus. The study was approved by the Research Ethics Boards of all universities involved; informed consent was obtained by all participants prior to survey administration. A demographic comparison of these resulting six cohorts is presented in Table 1.

**Table 1:** Participant Sociodemographic profile by province and rural/urban setting

	Manitoba		Ontario		Quebec		Total	Statistics; Effect size (χ^**2**^; Cramér’s V)^**a**^ or (***F*** statistics; Ω^**2**^ )^**b**^	***p*** Value
	Rural (***n***=248)	Urban (***n***=528)	Rural (***n***=79)	Urban (***n***=217)	Rural (***n***=197)	Urban (***n***=216)			
**Mean age in years** (standard deviation)^1. 3^	19.9 (1.6)	20.2 (1.9)	19.1 (1.2)	19.3 (1.5)	21.3 (1.5)	21.4 (1.6)		*F(5;1479)*=61.51; Ω^2^=0.17	<0.001***
**Sex** ^**2**^ **(%)**									
*Female*	84.6	70.3	70.9	74.5	82.6	76.2	75.8	χ^2^ (5)=25.05; V=0.13	<0.001***
*Male*	15.4	29.7	29.1	25.5	17.4	23.8	22.2		
**First year of postsecondary enrollment** ^2^ (%)	33.5	39.1	62.8	72.1	31.3	34.7	42.6	χ^2^ (5)=116.23; V=0.28	0.001**
**Full time course load** ^2^ (%)	94.4	92.0	94.9	92.6	92.8	94.4	93.1	χ^2^ (5)=2.40; V=0.043	0.752
**Past Year Academic Average** ^2^ (%)									
*Excellent (80-100%)*	59.5	53.2	38.0	46.8	61.9	59.7	54.6	χ^2^ (20)=66.07; V=0.21	<0.001***
*Very good (70-79%)*	31.6	29.0	38.0	28.7	28.4	25.9	29.3		
*Satisfactory (60-69%)*	4.0	8.0	19.0	8.8	3.0	9.3	7.6		
*Insufficient (<60%) / Uncertain*	2.4	5.3	3.8	9.7	4.6	1.4	4.7		
N*ot enrolled in courses*	2.4	4.5	1.3	6.0	2.0	3.7	3.8		
**Annual personal income** ^2^ (%)									
*$0*	5.7	14.8	24.1	29.6	8.6	16.2	15.6	χ^2^ (10)=91.77; V=0.18	<0.001***
*$1 - $15.000*	84.5	71.1	64.6	58.7	66.0	63.0	69.3		
*>$15.000*	9.8	14.1	11.4	11.7	25.4	20.8	15.4		
**Number of hours worked weekly** ^2^ (%)									
*Not employed*	32.7	29.5	55.7	48.1	38.1	38.9	36.6	χ^2^ (10)=41.56 V=0.12	<0.001***
*Fewer than 20 hours*	51.6	54.9	39.2	38.4	46.2	46.8	48.8		
*20 hours or more*	15.7	15.6	5.1	13.4	15.7	14.4	14.6		
**Race** ^2^ (%)									
*White*	7.8	54.0	87.0	38.2	95.8	84.3	67.1	χ^2^ (5)=399.28 V=0.24	<0.001***
*Black*	3.7	20.3	3.9	22.2	0.5	4.9	12.1		
*Asian*	3.3	9.7	5.2	13.7	1.0	4.9	7.2		
*First Nations and Métis*	15.4	6.0	0.0	0.0	1.6	2.0	5.2		
*Arabic*	0.0	6.4	1.3	14.6	0.0	1.0	4.7		
*Other*	0.8	3.5	2.6	11.3	1.0	2.9	3.8		
**Domestic / international student** ^2, 4^ (%)									
*D*o*mestic student*	96.4	75.9	93.5	78.8	87.3	83.8	87.3	χ^2^ (5)=67.72 V=0.21	<0.001**
*International student*	3.6	24.1	6.5	21.2	12.7	16.2	12.7		

^1^ *p* value calculated using ANOVA test

^2^ *p* value calculated using Chi-square or Fisher’s exact test

^3^All pairwise comparisons for ANOVA test using Bonferroni for the Age variable were statistically significant at 5 % except the comparison Urban Manitoba versus Rural Manitoba, Urban Ontario versus Rural Ontario, and Urban Québec versus Rural Québec

^4^Includes those whose country of citizenship is other than Canada and who travelled to study; excludes those who travelled from another Canadian province to study

^a^Chi-square (χ^2^*)*; Cramer’s V for Chi-square or Fisher’s exact tests

^b^Fisher (*F*) statistics; Ω^2^ for ANOVA test

*p < 0.05; **p < 0.01; ***p < 0.001

### Sample

Data was collected through convenience sampling. Each university’s research team recruited participants through an email sent to all students via their Students’ Union, inviting them to complete the online survey, or by administering the survey in classes where faculty professors agreed to provide class-time for survey completion. Of note, an effort was made to have adequate representation of years of study, programmes, and of various student profiles (ethnolinguistic primarily). Data collection occurred over 3 weeks in November 2018 (1 month post-RCL) and again over 3 weeks in February 2019. A total of 1496 students participated between both, either in 2018 and 2019 data collections (USB—529, BU—252, UO—297, UQAT—237, B’sU—181).

### Measures

The broad-ranging, 66-question survey, developed by the research team for a similar study in 2012 at USB and slightly modified (de Moissac et al. 2019), focused on academic and socioeconomic profiles, ethnolinguistic identity, mental health, substance use, sexual practices, road safety, and use of new technologies. Questions relating to risk-taking behaviours, including use of cannabis, alcohol, and illicit drugs (cocaine, heroin, etc.), as well as impaired driving, were inspired by the American College Health Survey (American College Health Association 2016). For example, questions pertaining to drug use were framed as “Have you ever used the following drug …”, with possible responses including “Never in my lifetime”, “Yes but not in past 12 months”, “Yes but not in past 30 days”, and “Used in past 30 days”. The survey was made available in print, and online through LimeSurvey, an online survey tool with Canadian data hosting.

### Statistical analyses

All statistical analyses were performed using SPSS version 24.0 for Windows (SPSS, Inc., Chicago, IL). Sociodemographic variables, substance use, and road safety practices were described as proportions for categorical variables, and as means and standard deviations for the age variable. Chi-square or Fisher’s exact tests, and ANOVA tests, were used to identify statistically significant differences between cohorts (Urban—Manitoba; Rural—Manitoba; Urban—Ontario; Rural—Ontario; Urban—Québec; Rural—Québec). The Ω^2^ for omnibus ANOVA for the age variable and the Cramér’s *V* for chi-squared tests for other variables were also computed to estimate effect sizes. To compare post-RCL substance use and related road safety behaviours between provinces and urban/rural settings, while adjusting for sociodemographic characteristics (i.e. age and gender), multivariate logistic binary regressions were performed. In all logistic regressions analyses, the outcome variable was coded “1” for “Yes” and “0” for “No”. The independent variables were grouped into two blocks: Block 1 included sociodemographic variables, and Block 2 included province, urban/rural setting, and their interaction. Due to the high number of sociodemographic variables, univariate logistic models with *p* ≤ 0.20 were tested to select the variables used in the multiple logistic regression, and a forward stepwise variable selection procedure was conducted as a first block. Enter procedure was used in Block 2. We derived adjusted odds ratios (AORs) at 95% confidence intervals (CIs) from the multivariable logistic regression models. Based on the criterion that the standardized residual (std. residual) is lower than three, outliers were checked. AORs used Manitoban cohorts, or urban cohorts, as references. For all comparisons, a significance level of 5% was the criterion for detecting a statistically significant effect. Collinearity statistics were conducted by using tolerance (< 0.1) for multiple linear regression models.

## Results

### Sociodemographic profiles

Sociodemographic profiles are presented by province and urban/rural contexts in Table 1. Statistically significant differences were observed in almost all sociodemographic variables (*p* < 0.001). Overall, cohorts’ mean age varied significantly according to province, with respondents in both Quebec cohorts representing the oldest respondents, while the youngest were in Ontario’s rural cohort. A large majority of participants was female, with this disparity being most significant in both Manitoba’s and Quebec’s rural cohorts, in which over 4/5 respondents identified as female. Approximately one third of respondents were in their first year of postsecondary studies, apart from Ontarian respondents, who were mostly in their first year. Except for the mostly first-year Ontarian cohorts, most respondents reported a full-time course load and excellent or very good academic averages, were employed, and reported personal annual income, with the highest earners (earning over $15,000 annually) found in both of Quebec’s cohorts. Roughly half of Ontarian respondents were unemployed, whereas this was the case for approximately a third of all other participants. In all three provinces, more international respondents, and White respondents, reported having lived in an urban context, compared to those who lived most of their life in a rural setting. The distribution of race variable is different between the six cohorts. Quebec’s rural cohort had the higher prevalence of white respondents (94.8%) while Ontario’s urban cohort had the low prevalence (38.2%).

### Substance use

Substance use prevalence for each cohort is reported in Table 2. Statistically significant differences were observed, with greater prevalence of substance use often associated to rural living. Manitoba’s and Ontario’s rural cohorts were found to report greater prevalence of recreational cannabis use throughout the subject’s lifetime compared to these province’s urban cohorts. However, prevalence of lifetime cannabis use in either Quebec cohorts is comparable (61.4% for rural and 58.2% for urban).

**Table 2:** Prevalence of recreational cannabis, alcohol, tobacco, and illicit drug use. by province and rural/urban setting

	Manitoba		Ontario		Quebec			Statistics; Effect size (χ^**2**^; Cramér’s V)	***p*** Value
	Rural (***n***=248)	Urban (***n***=528)	Rural (***n***=79)	Urban (***n***=217)	Rural (***n***=197)	Urban (***n***=216)	Total		
**Recreational cannabis use**									
**Lifetime** (%)	53.5	46.0	57.3	44.0	61.4	58.2	51.5	χ^2^ (5)=21.83 V=0.13	< 0.001**
**Past year** (%)	42.5	36.1	50.7	37.7	42.8	42.9	40.0	χ^2^ (5)=8.89 V=0.11	0.114
**Past month** (%)	32.9	25.6	32.0	27.1	22.9	34.1	28.2	χ^2^ (5)=10.21 V=0.07	0.069
**Three times or more in past month** (%)	14.1	12.8	17.6	12.6	14.5	21.4	14.6	χ^2^ (5)=9.30 V=0.08	0.098
**Medical cannabis use**									
**Lifetime** (%)	6.6	7.5	12.0	7.8	5.4	9.9	7.7	χ^2^ (5)=4.89 V=0.06	0.429
**Past year** (%)	5.3	5.4	6.7	5.3	2.4	6.6	5.2	χ^2^ (5)=3.74 V=0.05	0.588
**Past month** (%)	3.5	4.3	6.7	2.9	3.6	5.5	4.2	χ^2^ (5)=3.23 V=0.05	0.665
**Three times or more in past month** (%)	1.8	2.50	4.0	0.5	2.4	4.4	2.4	χ^2^ (5)=7.62 V=0.08	0.178
**Alcohol use**									
**Lifetime** (%)	95.2	82.2	96.0	75.1	96.4	94.0	87.5	χ^2^ (5)=77.48 V=0.24	< 0.001***
**Past month** (%)	84.7	64.7	82.7	53.7	83.9	75.3	71.2	χ^2^ (5)=80.90 V=0.25	< 0.001***
**Frequency of use in past month** (%)^a^									
*1 or 2 days*	30.9	33.8	30.6	40.0	34.8	38.0	34.4	χ^2^ (15)=13.62 V=0.12	0.554
*3 to 5 days*	30.4	34.1	37.1	27.3	26.2	22.6	30.0		
*6 to 9 days*	24.2	20.5	17.7	20.9	21.3	23.4	21.6		
*10 days or more*	14.4	11.7	14.5	11.8	17.7	16.1	13.9		
**Binge drinking (consuming 5 or more alcoholic beverage within 2 or 3 h)**									
**Lifetime** (%)	81.4	64.2	80.0	49.3	78.4	75.7	69.1	χ^2^ (5)=74.40 V=0.24	< 0.001***
**Past month** (%)	56.7	42.0	46.7	29.0	40.7	37.6	42.0	χ^2^ (5)=37.15 V=0.17	< 0.001***
**Frequency in past month** (%)^a^									
*1 or 2 days*	33.0	36.3	43.5	47.3	51.8	50.4	41.6	χ^2^ (15)=31.21 V=0.18	0.008**
*3 to 5 days*	36.1	36.6	33.9	27.3	23.4	25.5	31.7		
*6 to 9 days*	21.1	19.2	11.3	19.1	14.2	12.4	17.4		
*10 days or more*	9.8	7.9	11.3	6.4	10.6	11.7	9.3		
**Illicit drug use** ^**b**^									
**Lifetime** (%)	14.6	19.4	12.0	11.7	28.2	14.6	19.1	χ^2^ (5)=28.75 V=0.15	< 0.001***
**Past year** (%)	5.3	11.8	9.3	9.6	17.8	5.3	11.7	χ^2^ (5)=21.48 V=0.13	0.001**
**Past month** (%)	1.8	2.8	4.0	4.6	6.1	1.8	3.9	χ^2^ (5)=10.92 V=0.09	0.053

χ^2^: Chi-square

**p < 0.01; ***p < 0.001

^a^For users

^b^Includes cocaine, heroin, fentanyl, ecstasy, MDMA, methamphetamines, methadone, morphine, psilocybin, LSD, PCP, GHB, and ketamine

Similar trends were maintained when analysing self-reports of alcohol use throughout their lifetime. Past-month alcohol use remained significantly more prevalent among rural cohorts compared to their urban counterparts in all three provinces. Likewise, binge drinking, defined as consuming five or more alcoholic beverages within 2 or 3 h, was also significantly more prevalent among rural cohorts in Manitoba and Ontario, with these subjects reporting greater incidence of this behaviour both through their lifetime and in the previous month compared to their urban counterparts. Frequency of binge drinking was also highest among alcohol users in Manitoba. Finally, significantly more rural Quebec respondents also reported lifetime or past year illicit drug use, including cocaine, heroin, fentanyl, ecstasy, MDMA, methamphetamines, methadone, morphine, psilocybin, LSD, PCP, and ketamine. No statistically significant differences between the cohorts were observed for medical cannabis use (with prescription).

### Impaired driving

Prevalence of operating a motor vehicle after having consumed alcohol or cannabis (data limited to the 87% of respondents who held a valid drivers’ licence), and of riding as a passenger in a vehicle operated by an impaired driver (based on respondents’ subjective perception of intoxication), is presented in Table 3. While no significant differences between cohorts were observed in operating a motor vehicle under the influence of cannabis throughout the lifetime or in the past month, significant differences were observed pertaining to this behaviour when impaired by alcohol. Reports of alcohol-related impaired driving were significantly higher than reports of cannabis-related impaired driving in Manitoba and Quebec, but were similar in Ontario’s cohorts. Although significantly more rural respondents reported alcohol-related impaired driving through their lifetime, fewer respondents in Ontario (both rural and urban) reported doing so in the previous month, and no significant differences were observed in frequency of driving under the influence of cannabis. However, significant differences were observed for driving under the influence of alcohol, although this was also less reported by respondents in both of Ontario’s cohorts compared to all other cohorts.

As for polysubstance use-related impaired driving (alcohol, cannabis, and illicit drugs), significantly more rural respondents reported doing so in their lifetime in all provinces compared to their urban counterparts; this greater incidence among rural cohorts was maintained for reports of this behaviour in the previous month, save for Quebec’s rural cohort which reported this slightly less than their urban counterparts. Prevalence and frequency of driving while under the influence of illicit drugs was similar between cohorts. However, reports of riding as a passenger in a vehicle operated by an impaired driver (cannabis, alcohol, or illicit drugs) through their lifetime, in the previous month, or more than once in the previous month were less prevalent in Ontarian’s urban cohorts compared to all others.

### Multiple logistic regressions of substance use and impaired driving

Multiple logistic regressions analyses were conducted to explore the influence of the provincial and urban/rural living contexts on recreational cannabis use and driving under the influence of cannabis, adjusting for sociodemographic variables including age, gender, first year of postsecondary enrollment, and international/domestic student status. Collinearity was verified, revealing that no collinearity relationships exist in data (VIF < 2.7). Results and discussion relative to sociodemographic variables are beyond this paper’s scope.

**Table 3:** Driving or being a passenger while driver is under the influence of cannabis, alcohol, or illicit drugs by province and rural/urban setting

	Manitoba		Ontario		Quebec			Statistics; Effect size (χ^**2**^ ; Cramer’s V)	***p*** Value
	Rural (***n***=248)	Urban (***n***=528)	Rural (***n***=79)	Urban (***n***=217)	Rural (***n***=197)	Urban (***n***=216)	Total		
**Driving a motor vehicle under the influence:** ^**a**^									
**Cannabis.** Lifetime (%)	16.8	13.3	15.7	9.5	8.9	14.5	13.2	χ^2^ (5)=7.83 V=0.08	0.166
**Cannabis.** Past month (%)	7.8	6.4	11.4	4.7	3.8	9.2	6.8	χ^2^ (5)=7.57 V=0.08	0.182
**Alcohol.** Lifetime (%)	28.9	21.4	14.3	11.2	31.2	28.8	23.2	χ^2^ (5)=29.96 V=0.16	< 0.001***
**Alcohol.** Past month (%)	11.2	8.6	5.7	7.1	14.6	15.7	10.4	χ^2^ (5)=12.96 V=0.10	0.024*
**Illicit drugs**^**§**^**.** Lifetime (%)	3.5	3.8	7.1	4.80	3.8	7.2	4.5	χ^2^ (5)=4.98 V=0.07	0.418
**Illicit drugs**^**§**^**.** Past month (%)	0.0	1.9	4.30	2.4	1.3	1.3	1.6	χ^2^ (5)=8.09 V=0.08	0.151
**Cannabis, alcohol, or illicit drugs.** Lifetime (%)	34.3	26.0	21.4	15.0	35.3	32.2	27.8	χ^2^ (5)=26.50 V=0.15	< 0.001***
**Cannabis, alcohol, or illicit drugs.** Past month (%)	16.1	11.9	12.9	9.6	17.3	20.4	14.2	χ^2^ (5)=11.47 V=0.10	0.043*
**Frequency of driving under the influence in previous 30 days** ^**a**^									
**Cannabis** (%)									
*Never*	83.20	86.70	84.30	90.50	91.10	85.50	86.8	χ^2^ (15)=40.33 V=0.18	0.313
*Lifetime but not in past 30 days*	9.10	6.90	4.30	4.70	5.10	5.30	6.4		
*1-3 times in past 30 days*	6.00	3.60	7.10	3.00	3.20	7.20	4.6		
*4 times or more in the past 30 days*	1.70	2.90	4.30	1.80	0.60	2.00	2.2		
**Alcohol** (%)									
*Never*	71.10	78.60	85.70	88.80	68.80	76.8	71.20	χ^2^ (15)=17.09 V=0.12	< 0.001***
*Lifetime but not in past 30 days*	17.70	12.80	8.60	4.10	16.60	12.8	13.10		
*1-3 times in past 30 days*	10.30	7.10	4.30	4.70	12.70	8.9	14.40		
*4 times or more in the past 30 days*	0.90	1.40	1.40	2.40	1.90	1.5	1.30		
**Illicit drugs** ^**b**^ (%)									
*Never*	96.50	96.20	92.90	95.20	96.20	92.80	95.5	χ^2^ (5)=23.35 V=0.14	0.077
*Lifetime but not in past 30 days*	3.50	1.90	2.90	2.40	2.60	5.90	2.9		
*1-3 times in past 30 days*	0.00	0.70	2.90	2.40	1.30	1.30	1.3		
*4 times or more in the past 30 days*	0.00	1.20	1.40	0.00	0.00	0.00	0.0		
**Passenger in a motor vehicle driven by someone under the influence of cannabis, alcohol, or illicit drugs**									
**Lifetime (%)**	34.90	28.80	32.00	15.90	32.70	36.50	29.6	χ^2^ (5)=27.19 V=0.14	< 0.001***
**Past month (%)**	15.50	11.80	20.00	5.80	8.20	13.80	11.8	χ^2^ (5)=18.05 V=0.11	0.003**
**More than once in the past 30 days** (%)	9.70	7.00	13.30	4.30	2.90	7.40	7.0	χ^2^ (5)=13.94 V=0.10	0.016*

χ^2^, Chi-square

*p < 0.05; **p < 0.01; ***p < 0.001

^a^Among those who held a valid driver’s license

^b^As only a small proportion of participants reported driving a motor vehicle under the influence of illicit drugs. these data must be interpreted with caution

**Fig. 1:**
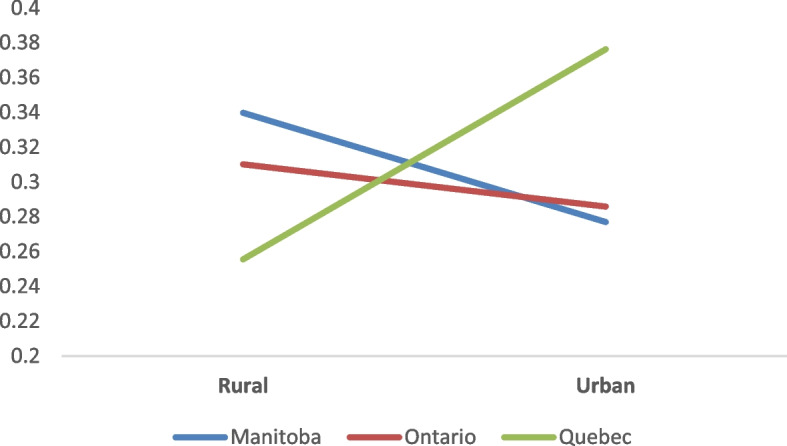
Probability of using recreational cannabis, past month

Adjusted odds ratios (AORs) and goodness of fit for recreational cannabis use are presented in Table 4. Quebec students were more likely to consume cannabis during their lifetime (AOR = 1.41, 95% CI [1.05, 1.90]) compared to the reference. Also, overall, rural participants were more likely to consume cannabis in the previous year compared to their urban counterparts. The significant interaction effect observed between provincial and urban/rural cohorts for cannabis use in the previous month suggests that the relation between cannabis use and living context differed between subjects in Quebec and those in the two other provinces. As shown in Fig. 1, Quebec’s students having lived mostly in urban contexts were more likely to use cannabis in the past month than those in rural contexts, whereas the opposite was observed in Manitoba and Ontario.

**Table 4:** Adjusted odds ratios (AOR) for recreational cannabis use

	AOR (95% CI)	*p* value	AOR (95% CI)	*p* value	AOR (95% CI)	*p* value
**Recreational cannabis use**	**Lifetime**		**Past year**		**Past month**	
Province						
Manitoba (Ref)	---------------		--------------		---------------	
Ontario	1.10 (0.82; 1.49)	0.522	1.22 (0.91; 1.63)	0.195	1.05 (0.71; 1.55)	0.825
Quebec	**1.41 (1.05; 1.90)**	0.022*	1.26 (0.96; 1.65)	0.101	**1.58 (1.06; 2.33)**	0.023*
Spent the majority of their life in						
Urban setting (Ref)	---------------		---------------		---------------	
Rural setting	1.25 (0.98; 1.60)	0.072	**1.32 (1.04; 1.67)**	0.024*	1.34 (0.93; 1.94)	0.115
Interaction (province × spent the majority)						
Ontario—rural setting					0.84 (0.41; 1.69)	0.619
Quebec—rural setting					**0.42 (0.23; 0.78)**	0.006**
**Goodness of fit**						
* χ*^2^(df; *p*)	91.20 (11;* p* < 0.001)		30.63 (9; *p* < 0.001)		61.04 (15; *p* < 0.001)	
* χ*^2^ Hosmer and Lemeshow (df; *p*)	12.13 (8; *p* = 0.145)		9.99 (8; *p* = 0.266)		6.96 (8; *p* = 0.541)	
* R*^2^ de Nagelkerke	0.091		0.032		0.067	
Correct percentage classifying (%)	61.8		61.4		72.3	

Data presented as adjusted odds ratios (with confidence interval of 95%) using logistic binary regression adjusted for sociodemographic variable (Ex. age, gender, first year of postsecondary enrollment. international/domestic students)

*AOR* adjusted odds ratio, *CI* confident interval, *Ref* reference group

*p < 0.05;  **p < 0.01

**Fig. 2:**
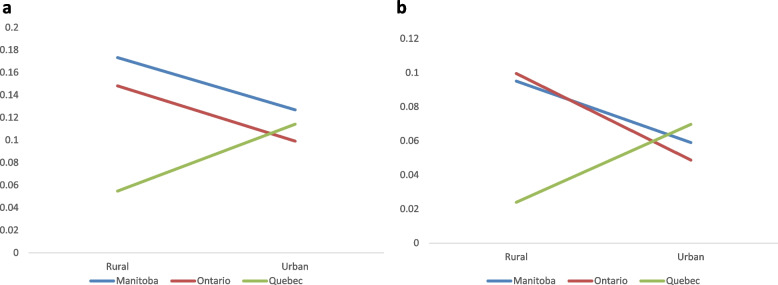
**a** Probability of operating a motor vehicle after using cannabis, lifetime. **b** Probability of operating  motor vehicle after using cannabis, last month

AORs relating to road safety were assessed and are presented in Table 5. Although AORs investigating the main effects of provincial or urban/rural contexts on operating a motor vehicle after using cannabis (lifetime and past month) yielded no significant differences, a significant interaction effect was observed. This interaction suggests that the relation between cannabis-impaired driving and urban/rural contexts for Quebec students differed from that of Manitoban and Ontarian respondents (Fig. 2a, b). Quebec’s urban cohort was more likely to report cannabis-impaired driving, both through respondents’ lifetime and in the previous month, while the opposite was observed in Manitoba and Ontario. Pertaining to the broader behaviour of driving under the influence of alcohol, or under the influence of cannabis, alcohol, or illicit drugs, a statistically significant difference was observed only as reported throughout the participant’s lifetime, but not in the past month. This result suggests that Ontarian respondents had lower odds of driving under the influence of alcohol (AOR = 0.62, 95% CI [0.39, 0.98]) and under the influence of cannabis, alcohol, or illicit drugs (AOR = 0.66, 95% CI [0.44, 0.99]), compared to Manitoban respondents. Also, participants from rural settings were more likely to report alcohol-impaired driving, and driving while under the influence of alcohol, cannabis, or illicit drugs (AOR = 1.46, 95% CI [1.08, 1.96]; AOR = 1.46, 95% CI [1.11, 1.92] respectively) than respondents from urban centres.

**Table 5:** Adjusted odds ratios (AOR) for driving under the influence

	AOR (95% CI)	***p*** Value	AOR (95% CI)	***p*** Value
**Operating a motor vehicle after using cannabis** ^**a**^	**Lifetime**		**Past month**	
Province				
Manitoba (Ref)	---------------		---------------	
Ontario	0.76 (0.41 ; 1.39)	0.368	0.82 (0.35 ; 1.89)	0.637
Quebec	0.89 (0.51 ; 1.55)	0.672	1.20 (0.58 ; 2.45)	0.629
Spent the majority of their life in				
Urban setting (Ref)	---------------		---------------	
Rural sitting	1.44 (0.91 ; 2.28)	0.119	1.67 (0.87 ; 3.20)	0.120
Interaction (Province x Spent the majority …)				
Ontario - Rural setting	1.10 (0.41 ; 2.91)	0.855	1.29 (0.37 ; 4.55)	0.694
Quebec - Rural setting	**0.31 (0.13 ; 0.78)**	0.012*	**0.20 (0.05 ; 0.73)**	0.015*
**Goodness of fit**				
χ^2^(df; p)	29.31 (10; *p* = 0.001)		43.98 (11; *p* < 0.001)	
χ^2^ Hosmer and Lemeshow (df; p)	8.62 (8; *p* = 0.375)		11.11 (8; *p* =0.195)	
R^2^ de Nagelkerke	0.047		0.097	
Correct percentage classifying	86.8		93.3	
**Operating a motor vehicle after using alcohol** ^**a**^	**Lifetime**		**Past month**	
Province				
Manitoba (Ref)	---------------		---------------	
Ontario	**0.62 (0.39 ; 0.98)**	0.041*	0.75 (0.41 ; 1.39)	0.359
Quebec	0.90 (0.64 ; 1.27)	0.545	1.28 (0.81 ; 2.03)	0.285
Spent the majority of their life in				
Urban setting (Ref)	---------------		---------------	
Rural setting	**1.46 (1.08 ; 1.96)**	0.013*	1.25 (0.83 ; 1.87)	0.288
**Goodness of fit**				
χ^2^(df; p)	86.51 (7; *p* < 0.001)		45.55 (8; *p* < 0.001)	
χ^2^ Hosmer and Lemeshow (df; p)	5.50 (8; *p* = 0.703)		12.49 (8; *p* = 0.130)	
*R*^2^ de Nagelkerke	0.11		0.080	
Correct percentage classifying	76.6		89.5	
**Operating a motor vehicle after using cannabis, alcohol, or illicit drugs** ^**a**^	**Lifetime**		**Past month**	
Province				
Manitoba (Ref)	---------------		---------------	
Ontario	**0.66 (0.44 ; 0.99)**	0.042*	0.79 (0.48 ; 1.30)	0.356
Quebec	0.82 (0.59 ; 1.14)	0.248	1.07 (0.71 ; 1.61)	0.750
Spent the majority of their life in				
Urban setting (Ref)	---------------		---------------	
Rural setting	**1.46 (1.11 ; 1.92)**	0.008**	1.32 (0.92 ; 1.88)	0.131
**Goodness of fit**				
χ^2^(df; p)	68.29 (6; *p* <0.001)		61.22 (10; *p* < 0.001)	
χ^2^ Hosmer and Lemeshow (df; p)	5.15 (7; *p* =0.642)		15.35 (8; *p* = 0.053)	
*R*^2^ de Nagelkerke	0.084		0.092	
Correct percentage classifying	72.0		85.7	
**Passenger in a motor vehicle driven by someone under the influence of cannabis, alcohol, or illicit drugs**	**Lifetime**		**Past month**	
Province				
Manitoba (Ref)	---------------		---------------	
Ontario	**0.53 (0.34 ; 0.82)**	0.005**	**0.47 (0.24 ; 0.94)**	0.032*
Quebec	1.31 (0.90 ; 1.93)	0.162	1.28 (0.75 ; 2.18)	0.374
Spent the majority of their life in				
Urban setting (Ref)	---------------		---------------	
Rural setting	1.21 (0.85 ; 1.72)	0.287	**1.64 (1.03 ; 2.61)**	0.037*
Interaction (Province x Spent the majority …)	---------------		---------------	
Ontario - Rural sitting	**2.18 (1.06 ; 4.48)**	0.033*	**2.68 (1.01 ; 7.10)**	0.048*
Quebec - Rural setting	0.64 (0.36 ; 1.13)	0.119	**0.38 (0.16 ; 0.90)**	0.027*
**Goodness of fit**				
χ^2^(df; p)	61.38 (10; *p* <0.001)		44.26 (11; *p* < 0.001)	
χ^2^ Hosmer and Lemeshow (df; p)	10.99 (8; *p* =0.202)		2.93 (8; *p* = 0.939)	
*R*^2^ de Nagelkerke	0.065		0.065	
Correct percentage classifying	70.9		88.4	

Data presented as adjusted odds ratios (with confidence interval of 95%) using logistic binary regression adjusted for sociodemographic variable (Ex. age, gender, first year of postsecondary enrollment, international/domestic students)

*AOR* Adjusted odds ratio, *CI* Confident Interval, *Ref* Reference group

* *p* < 0.05; ** *p* < 0.01

^a^among those who held a valid driver’s license

**Fig. 3:**
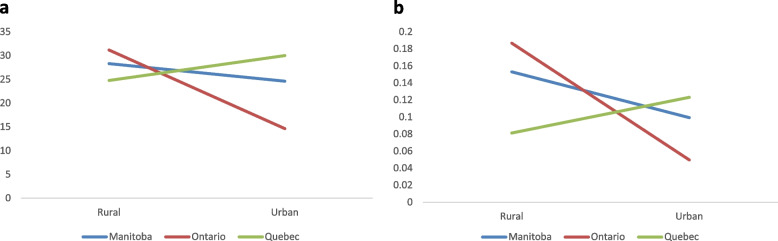
**a** Probability of being passenger in a motor vehicle driven by someone under the influence of cannabis, alcohol, or illicit drugs, lifetime. **b** Probability of being passenger in a motor vehicle driven by someone under the influence of cannabis, alcohol, or illicit drugs, past month

Significant interaction effects were also observed for being passenger in a motor vehicle operated by an impaired driver both in their lifetime and in the previous month. As shown in Fig. 3a, Ontario’s urban respondents were least likely to report this behaviour in their lifetime. In the previous month, Manitoban and Ontarian rural respondents had greater odds of reporting this behaviour compared to their urban counterparts, while the opposite was observed in Quebec’s cohorts, where rural respondents had lower odds of reporting this behaviour in the previous month compared to their urban counterparts.

## Discussion

This paper presents self-reported substance use and road safety behaviours of EAs (18 to 24 years of age) enrolled in one of five Canadian postsecondary institutions in Manitoba, Ontario, and Quebec, shortly post-RCL, allowing to compare provincial and rural/urban trends. Statistically significant differences were observed between cohorts in almost all measures, corroborating previous reports of differences in interprovincial (Rotermann 2020) and rural/urban living contexts in other populations (Azagba et al. 2020; Derefinko et al. 2018; Pirie and Simmons 2014). Of note, trends relating to substance use and impaired driving are similar between Ontario’s and Manitoba’s cohorts, with greater prevalence of these behaviours observed among those with rural upbringing as compared to students from urban centres. These trends differ, however, in Quebec, where urban respondents were more likely to engage in substance use and hazardous substance-related driving practices compared to respondents from rural settings. Data provide valuable insights for the development of targeted prevention strategies and initiatives geared to promoting the health and safety of postsecondary students in these three provinces.

### Substance use trends

The first aim of this paper was to compare substance use trends between rural/urban and provincial context. Although, cannabis users in all cohorts largely reported consuming cannabis recreationally as opposed to medically (Statistics Canada 2019d); alcohol use remained significantly more prevalent than cannabis use in all cohorts (American College Health Association 2016; Gueye et al. 2019; Statistics Canada 2017). Nationally, prevalence of past-3-month cannabis use by EAs was 33.2% during the present study’s timeframe (Statistics Canada 2019b), which is comparable to past-month use in this sample, which ranged from 22.9% of rural Quebec respondents to 34.1% of this same province’s urban respondents. As for postsecondary students specifically, the American College Health Association (ACHA) placed pre-RCL (2016) prevalence of past-month cannabis use by Canadian students of all ages at 17.9% (American College Health Association 2016). Let us caution however that RCL only allowed for those aged 19 years or over to consume cannabis recreationally in Manitoba and Ontario, whereas 18-year-olds could legally consume in Quebec at the time of this study; this may have impacted results among the present 18-to 24-year-old sample.

Past-month cannabis use was significantly more prevalent in rural compared to urban cohorts in Manitoba and Ontario; however, this trend was reversed in Quebec. Furthermore, Manitoban and Ontarian urban cohorts tended to consume fewer substances than their rural counterparts, as observed for alcohol consumption and almost all other measures. Interestingly, AORs conducted to compare provincial and living contexts on recreational cannabis use reveal that in Quebec, respondents were more likely to report lifetime recreational cannabis use compared to the reference. Furthermore, rural respondents were found to be more likely to report recreational cannabis use compared to the urban reference for past-year use, but not so for lifetime or past-month use.

These findings may help elucidate the divided literature examining rural/urban differences in cannabis consumption among youths and postsecondary students, highlighting some geographical factors which may cause significant disparity across Canada. Indeed, although some report negligible differences between Canadian populations (Pirie and Simmons 2014), others have observed higher prevalence of consumption in urban compared to rural populations (Coughlin et al. 2019; Derefinko et al. 2018). Hence, our data suggest that the provincial variable is vital to better understand trends relating to these behaviours and to design more effective substance misuse prevention and intervention initiatives. Other factors, specific to young adults, should also be considered: a sense of belonging and connectedness to a larger community, positive relationships with adults, a sense of personal security, and rigorous family and peer supports are efficacious protective factors against substance misuse among this population and could further guide future interventions (Tam 2018; Zuckermann et al. 2020).

It is important to note that present data were collected in the first months of RCL in Canada. During this time, storefront and home delivery cannabis sales operating in Manitoba (Province of Manitoba 2018) would have provided for greater licit access to cannabis and greater opportunity to maintain anonymity, compared to Ontario and Quebec, where purchases were limited to online ordering (Legislative Assembly of Ontario 2018; Province of Quebec 2020). However, data suggest that cannabis use trends among Canadian postsecondary students seem to supersede provincial differences in legislation and access, as urban dwellers in Manitoba, who would have had greater access to licit cannabis sources, reported lower prevalence of past-month consumption than most other cohorts, where licit cannabis access was relatively more limited. Indeed, as shown previously, cannabis-related legislation appears to be largely inconsequential on EA recreational cannabis use (Gueye et al. 2021; Midgette and Reuter 2020; Simons-Morton et al. 2010; Smart and Pacula 2019). This observation may reflect the normalization of cannabis use, or greater discourse surrounding cannabis, which took place within the Canadian society for some time pre-RCL (Canada 2018; Zuckermann et al. 2020), and may have favoured the development of more permissive social attitudes relating to cannabis use. It may also be possible that users maintained illicit channels for sourcing cannabis, although Canadians seem to be gradually shifting from illicit to licit sources (Wallingford et al. 2019).

### Trends for impaired driving

The second aim of this paper was to measure and compare prevalence of impaired driving and riding in a vehicle operated by an impaired driver, among postsecondary student populations, as impairment significantly increases the risk of traffic-related injury or death (Brubacher et al. 2020). Although no statistically significant differences were observed between cohorts, past-month cannabis-impaired driving was reported by no fewer than 11.4% of Ontario’s rural cohort, while all other cohorts were below 10% for this measure. Encouragingly, present prevalence of driving after consuming cannabis in all surveyed timeframes is well below the national average for 18-to 24-year olds, of whom 16.0% having reported done so since RCL, and also well below provincial averages (17.6% in Manitoba, 12.4% in Ontario, 13.7% in Quebec; Rotermann 2020). However, observed prevalence is greater than the 6% (Manitoba), or 4% (Ontario and Quebec), of 20-to-24-year-olds who reported cannabis-impaired driving in 2012 (Wettlaufer et al. 2017). Hence, reports of this hazardous behaviour among this demographic appear to be changing and should be closely monitored. Also, the student composition of this sample must be front of mind, as fewer students tend to report impaired driving than their non-student EA peers (Huỳnh et al. 2022).

Prevalence of driving under the influence of alcohol remained higher than that of cannabis in this sample. This may seem surprising as 16-to-30-year-old Canadians tend to appraise the risk of driving under the influence of alcohol (both risk of injury and risk of legal ramifications) to be significantly greater than that of driving after consuming cannabis (Goodman et al. 2020). Gender and age may have been confounding variables, as they have been found to be significant predictors of cannabis-impaired driving, with men and younger individuals at greater risk (McDonald et al. 2021; Rotermann 2020; Wallingford et al. 2019). However, one Canadian study found that this gender-based disparity in impaired driving may have leveled through the COVID-19 pandemic, with men and women reporting this behaviour at similar rates (Vanlaar et al. 2021). This supports the need for continued investigation of this phenomenon, as trends change over time.

Impaired driving appears to be greater among students in this sample with rural upbringing, especially in Manitoba and Ontario. Greater vehicle ownership and licensing among rural residents may explain some of these rural/urban differences, as personal vehicles are the sole means of transportation for most rural residents (Statistics Canada 2019e), whereas urban residents could choose alternate modes of transportation when intoxicated, such as public transit, active transportation, taxis, or ride sharing services. Interestingly, the likelihood of driving after consuming cannabis among this population seemed unchanged post-RCL in Canada (Rotermann 2020), and drivers in Manitoba, Ontario, and Quebec report the lowest prevalence of impaired driving out of all provinces (Perreault 2015).

The postsecondary-only composition of this sample is important to consider when comparing present data to larger Canadian data collected among EAs. Canadian EAs attending school, either full-time or while working, are significantly less likely to report driving after consuming cannabis compared to their peers who do not attend school: the latter represented 70.0% of all EA respondents reporting this behaviour in a 2018–2019 Canadian study (Huỳnh et al. 2022). This suggests attendance at a postsecondary institution, even if not full-time, may serve as a protective factor against driving while intoxicated, and prevalence within the larger population may be far greater than that observed in this sample. Also, considering present data were collected in the wake of RCL, it seems this permissive legislation did not bear an impact on trends relating to driving while under the influence of cannabis (Rotermann 2020; Statistics Canada 2019a) or on emergency room admissions for cannabis-related traffic injuries (Callaghan et al. 2021).

### Trends pertaining to riding in a vehicle operated by an impaired driver

Regarding riding as a passenger in a vehicle operated by an intoxicated driver, national data collected during a comparable timeframe reveals 11.9% of 18-to-24-year-old respondents had travelled in a vehicle operated by someone who had consumed cannabis within the previous 2 h (Rotermann 2020). Rural Ontarian respondents were the least likely to report having travelled in a vehicle operated by an intoxicated driver (either cannabis, alcohol, or illicit drugs) out of all cohorts. Interestingly, pre-RCL Canadian data from 2014 to 2015 revealed Manitobans to be least likely, compared to Ontarians and Quebecers, to report riding in a car with a driver intoxicated to alcohol, but most likely to report doing so for cannabis (Minaker et al. 2017). While present AORs reveal significant trends, whereby the Ontarian cohorts were far less likely to report riding in a vehicle operated by an impaired driver than the Manitoban reference, no main effect of rural or urban context is observed. However, significant interaction effects were observed, suggesting rural Ontarian respondents were less likely to report this behaviour than the Manitoban reference, while rural Quebecers approximately half as likely to report doing so.

### Limitations and future research

This study purveys valuable insights which will inform policymakers and service providers at academic, healthcare, and governmental levels. However, some limits must be kept front of mind when interpreting present results. As institutions selected for this study are of small or medium-size (UO excepted), findings may only be generalizable to student populations in similar-sized campuses and not to all emerging adult populations. Moreover, as varying numbers of participants were recruited in each institution due to convenience sampling and use of heterogeneous recruitment strategies, all demographics were not equally represented. A significant disparity between the number of male and female respondents was also observed. Although this disparity was expected, as female students are more likely to participate in such studies (American College Health Association 2016) and often outnumber male students in Canadian universities (Turcotte 2011), this may have also impacted results, as gender is a significant predictor of substance use and impaired driving. However, in response to this sample’s gender disparity, we controlled for age, gender, and other sociodemographic variables when calculating AORs. Furthermore, convenience sampling and the cross-sectional nature of this study must be considered, as these may have biased findings and preclude the postulation of any causal interpretation. The innate subjective nature of self-reported data must also be kept in consideration. The questionnaire used in this study was intended to survey mental health and risk-taking behaviours: specific questions pertaining to participants’ licit/illicit sourcing of substances were not included.

Future studies, using random sampling and measures targeted to substance use, will further elucidate trends and could attempt to establish causal variables. Also, given the significant impact of provincial context found in this study, future studies could also focus on cultural differences between provinces which may explain interprovincial disparities. Furthermore, the continued study of substance-related trends will offer insights on this evolving reality, especially in the wake of the COVID-19 pandemic.

## Conclusion

This cross-sectional study contributes to the literature by addressing substance use and impaired driving trends among Canadian postsecondary students EAs in the wake of RCL, allowing for interprovincial and rural/urban comparisons. Significant differences were observed between all six cohorts in almost all measures, revealing that both urban/rural and provincial context must be taken into account in tandem to better appreciate trends. Respondents in Quebec and in rural settings reported significantly greater prevalence of recreational cannabis use (lifetime and past year respectively), and rural respondents were significantly more likely to report driving while impaired by cannabis, alcohol, or drugs. The relation between cannabis use in the previous month, cannabis-impaired driving (lifetime and past month), and living context differed between cohorts. Taken together, present data suggest that caution should be taken against the postulation of broad rural/urban generalizations, as these differ significantly according to province of residence. Results also suggest the need for targeted prevention campaigns tailored to the substance and to provincial and rural/urban populations. However, the context is rapidly shifting: cannabis supply and quality issues commonplace in all three provinces at the time of this study may have mitigated use among all cohorts. Investigation should be pursued as markets, legislation, and consumer trends stabilize.

## Data Availability

The datasets generated and/or analysed during the current study are not publicly available due to ongoing data treatment but are available from the corresponding author on reasonable request.
